# Epithilial Sowing—A New Method of Skin-Grafting

**Published:** 1897-02

**Authors:** 


### EPITHELIAL SOWING: A NEW METHOD OF SKIN-GRAFTING.

Von Mangoldt, of Dresden (*La Semaine Med.*, XV., 1895, p. 520), employs the following method of skin-grafting: First, he selects the part from which the grafts are to be removed, preferably the inner or outer surface of the arm; then, after thoroughly cleansing and antisepticising the spot, the razor is sterilized and held perpendicular to the skin, the epidermis being scraped away until the papillary layer is reached. In this way a magma is obtained, being composed of extravasated blood and epithelial cells, which is placed upon and pressed into the part to be treated. At times the author first scarifies the part to make sure of adherence. After the foregoing, strips of adhesive dressing are placed over the part. This method, to which the author has given the name of "epithelial sowing," is said to have advantages over the Thiersch method in that no pockets of necrotic tissue are closed in by the new formed skin. After the fifth day the dressing is changed every two days, and the wound gently irrigated with sterile and warmed normal salt solution, and towards the end of the third week the surface shows a normal appearance.—*Philadelphia Polyclinic.*

